# Can Handheld Thermal Imaging Technology Improve Detection of Poachers in African Bushveldt?

**DOI:** 10.1371/journal.pone.0131584

**Published:** 2015-06-25

**Authors:** Adam G. Hart, Richard N. Rolfe, Shantelle Dandy, Hannah Stubbs, Dougal MacTavish, Lynne MacTavish, Anne E. Goodenough

**Affiliations:** 1 School of Natural and Social Sciences, University of Gloucestershire, Cheltenham, United Kingdom; 2 Mankwe Wildlife Reserve, Rustenburg, Northwest Province, South Africa; University of Hawaii at Manoa, UNITED STATES

## Abstract

Illegal hunting (poaching) is a global threat to wildlife. Anti-poaching initiatives are making increasing use of technology, such as infrared thermography (IRT), to support traditional foot and vehicle patrols. To date, the effectiveness of IRT for poacher location has not been tested under field conditions, where thermal signatures are often complex. Here, we test the hypothesis that IRT will increase the distance over which a poacher hiding in African scrub bushveldt can be detected relative to a conventional flashlight. We also test whether any increase in effectiveness is related to the cost and complexity of the equipment by comparing comparatively expensive (22000 USD) and relatively inexpensive (2000 USD) IRT devices. To test these hypotheses we employ a controlled, fully randomised, double-blind procedure to find a poacher in nocturnal field conditions in African bushveldt. Each of our 27 volunteer observers walked three times along a pathway using one detection technology on each pass in randomised order. They searched a prescribed search area of bushveldt within which the target was hiding. Hiding locations were pre-determined, randomised, and changed with each pass. Distances of first detection and positive detection were noted. All technologies could be used to detect the target. Average first detection distance for flashlight was 37.3m, improving by 19.8m to 57.1m using LIRT and by a further 11.2m to 68.3m using HIRT. Although detection distances were significantly greater for both IRTs compared to flashlight, there was no significant difference between LIRT and HIRT. False detection rates were low and there was no significant association between technology and accuracy of detection. Although IRT technology should ideally be tested in the specific environment intended before significant investment is made, we conclude that IRT technology is promising for anti-poaching patrols and that for this purpose low cost IRT units are as effective as units ten times more expensive.

## Introduction

Illegal hunting is a major wildlife problem in many regions of the world. A key driver of illegal hunting (hereafter referred to as poaching) is increasing urban expansion, which causes human encroachment of wildlife areas, elevated pressure on natural resources, and an increasing demand for bushmeat [1–2]. Subsistence hunting, where animals are taken to satisfy immediate local demand for food, still exists, but poaching is increasingly undertaken to supply more commercially-organised demand for bushmeat [3]. This demand is both within the country of origin and, increasingly, internationally (e.g., an estimated five tonnes per week is imported into Paris, from where it is distributed to other sites across Europe [4]).

Despite a worrying increase in the international bushmeat trade, poaching becomes far more overtly commercialised, and normally far more unsustainable, when animals are killed for hunted commodities other than meat [5]. Well-known international examples are tigers [6] and sun bears [7] illegally hunted for use in traditional medicines, alligators and crocodiles that are poached for skins [8], and the capture of live individuals of many species, especially reptiles, monkeys, and exotic birds, for the pet trade [9]. Commercial poaching of many iconic mammals within Africa has been substantial and is increasing in some cases. For example, African elephants are still killed for the illegal ivory trade [10–12], while endangered pangolins are subject to extreme poaching pressure for their scales [13–14]. There has also been an enormous rise in the amount of rhinoceros (white and black) poaching in recent years. The African black rhino is listed as critically endangered by the IUCN with the global population declining by 96% in the 35 years between 1970 and 1992, and has become extirpated in large parts of its former range, with one sub-species, *Diceros bicornis longipes*, now extinct [15]. Overall, the poaching of such animals for tusks, scales, horns and other commodities supply illegal–but highly lucrative–international markets with strong links to illegal trade (i.e. trade outside CITES) and organised crime [16–18]. Consequently, poaching is a significant problem both for long-term sustainability of animal populations and for those trying to control it.

Record levels of funding are being invested in anti-poaching measures as part of the “war on poaching” [19]. The type of anti-poaching measures undertaken, and their effectiveness, varies greatly with local conditions, traditions, laws, and resource availability. Foot and vehicle patrols are still the most commonly used anti-poaching methods, acting as a deterrent as well as helping to catch poachers. Recently, however, more technological solutions have been sought to complement these patrols and improve both detection and deterrence. This includes the use of unmanned aerial vehicles (UAVs, or drones), which have been tested for anti-poaching controls for rhinoceros in South Africa [20]. Such technology has had some promising results but there are important logistical limitations (including battery life) and, critically, financial constraints. Another approach involves camera trapping, but while arrests of poachers have been made using evidence from camera trap footage, such technology is not generally preventative [21]. In 2014, Banzi *et al*. proposed the use of large animals, such as elephants, as mobile biological sensors (MBS). The animals act as platforms for sensory technology that include visual and infrared cameras and GPS, which allows movements to be monitored and information regarding classic panic movement to be fed back to rangers [22]. This method, however, is costly and has practical issues–fitting the sensors requires training and batteries require changing–both of which cause disruption to animals and risk to rangers. One of the most promising technologies that could be used to complement anti-poaching patrols is thermal imaging. Thermal imaging is a particularly promising possibility as the technology can be used on traditional foot and vehicle patrols and is useful for both detecting poachers (including at night) and for monitoring potential animal targets.

Thermal imaging, or infrared thermography (IRT), is an imaging technology that can remotely sense the temperature of objects (including animals) with a sensor sensitive to infra-red wavelengths that are emitted and reflected from that object. Such technology has found many uses in biological and ecological studies [23] including detecting nocturnal, cryptic and burrowing species [24–25], surveying and monitoring mammals and birds [26], detecting injuries and infectious diseases [27], studying plant physiological processes [28] and in studies of reproductive biology [29]. IRT is especially useful for detecting mammals because individuals are typically warmer (sometimes considerably so) than their surroundings, often allowing them to be readily detected even in complete darkness [30–31]. As a mammal, humans also provide a strong thermal signature, although this can be masked with insulating clothing. Accordingly, many military and police applications of IRT have been developed, for example, detecting the exact locations of people in dangerous or covert situations and studying the movement and behaviour of suspects and captives in hostage situations [32]. Thermal imaging is a potentially attractive anti-poaching technique since much poaching activity, including setting/checking snares and hunting with dogs, occurs at night, which makes detecting poachers especially difficult and dangerous.

Increasing commercial interest in IRT, especially for surveying buildings, and a growing awareness of the range of other potential uses of the technology have led to the development of small but sensitive handheld devices (resembling police radar guns or large digital cameras) [32] with a concomitant decrease in price. Expensive, UAV-mounted IRT devices have already been used in anti-poaching campaigns (e.g. [20]), but the smaller size, ruggedness, ease of use, and lower price of recently-developed handheld devices suggest that IRT technology is within the grasp of smaller wildlife reserve and game farm management teams for foot patrols. That said, IRT technology is unlikely to be a single solution to the problem of detecting human intruders. Many habitats where poaching is a significant problem are likely to be thermally complex, and the heat signature given off by a human, even if not masked by posture or clothing, could be difficult to detect in field conditions.

Given that IRT is still relatively expensive, its effectiveness in detecting poachers in realistic field conditions needs to be tested before its adoption as an anti-poaching measure can be recommended. Here, we test the hypotheses that: 1) the use of IRT will increase the distance over which a poacher hiding in African scrub bushveldt can be detected relative to a conventional flashlight; and, 2) any increase in effectiveness is related to the cost and complexity of the equipment by comparing comparatively expensive (22000 USD) and relatively inexpensive (2000 USD) IRT devices.

## Methods

The study was undertaken in May 2014 at the Mankwe Wildlife Reserve, a 4750 ha privately-owned reserve situated in the North West Province of South Africa (centred on S 25.24963; E 27.32148), approximately 5km east of Pilanesberg National Park. The reserve is managed by two of the co-authors (LM and DM) and the work undertaken required no permits and did not include the sampling of any species, protected or otherwise. The reserve hosts a range of species including numerous large mammals. Subsistence and bushmeat poaching, as well as poaching of rhino for their horn as part of an organised crime network, is an on-going and increasing problem. Frequent foot patrols and fence drives, together with skilled trackers on the ground, are the principles of the current anti-poaching strategy. As most poaching at Mankwe takes place at night during a full moon (pers. comm. L and D Mactavish (co-authors)) the fieldwork study period was selected to coincide with these conditions.

### IRT devices and optimisation

To test the effectiveness of high and low priced IRT devices, a FLIR T620 (ca. 22000 USD) and a FLIR i7 (2000 USD) were used. These devices, and alternative devices that are similar in specification, are widely used in a range of biological applications that include surveying nocturnal mammals [33]. The FLIR T620 had a resolution of 640X480 pixels with a 30 Hz infrared detector. The measurable temperature range was -40 to 650°C with a thermal sensitivity of 0.04°C. The FLIR i7 had a resolution of 140X140 pixels with a 9 Hz infrared detector. The measurable temperature range was 20 to 250°C with a thermal sensitivity of 0.1°C.

A pilot study showed that both devices were capable of detecting mammals, including clothed humans, as hot spots in African bushveldt field conditions. Both IRT devices were optimised for detection of humans. The FLIR i7 was set to a monochrome colour palette and an isotherm was set at 8°C above the temperature of the surrounding bush, such that anything above this temperature showed up onscreen as red. The FLIR T620 was set to lava colour palette with an isotherm set at the same temperature as on the i7; in this case anything above the isotherm temperature appeared on-screen as green. The colour palettes were chosen from the range available following a pilot study and were selected to show a human as clearly as possible at similar distance ranges to those used in the main study, as judged by the authors (note that the i7 did not have the lava colour palette). The actual setting of the isotherm varied between and within nights according to local temperature changes, but was always between 12°C and 17°C. The pilot study showed that setting the isotherm *≤*8°C resulted in rocks appearing as hotspots, while setting it *≥*8°C resulted in humans becoming less visible as only faces were warm enough to show up (other areas of skin being covered with clothing).

### Study design

Searching for poachers at night and on foot is dangerous and slow. On the study site, such a search would only be undertaken if there was good reason to suspect a poacher was in a particular location. To simulate this situation, the study design employed a target poacher (henceforth referred to simply as target) hidden in a defined search area, with informed searchers looking for the target within that area.

An adult male (co-author RNR) was selected to be the target for the duration of the study. His participation in this aspect of the study was entirely voluntary and his authorship indicates full informed consent. He was dressed in full length, dark bush-coloured, clothing and wore a green hat. The target’s clothing was selected to be similar to that typically worn by poachers in the study location and the authenticity of the target’s appearance was confirmed by authors D and L MacTavish who are experienced in anti-poaching management in the study location.

To set a sensible detection distance, a series of photographs was taken at 10m intervals of the target standing in grassland (mean sward height of 1 m) using the FLIR T620 IRT two hours after sunset. This showed that it was possible for an experienced user to detect the poacher at 80m. Accordingly, a transect of 120m was marked out along a straight track. There were five target locations in the bush on one side of the transect. All locations were a perpendicular distance of 20m from the transect, at points 120m, 110m, 100m, 87m, and 74m along the transect. The distance between any point along the track and each hiding location could be calculated trigonometrically based on the distance travelled from the start point with all hiding locations being at least 77m from the start point.

The study area was representative medium density grassland dominated by, in order of abundance, broad curly leaf (*Eragrostis rigidor*), spear grass (*Heteropogon contortus*), common finger grass (*Digitaria eriantha*), cat’s tail (*Perotis patens*), natal red top (*Melinis repens*), wool grass (*Anthephora pubescens*) with a mean sward height of 0.8m and was approximately level topographically, with a low density (2–3 per square metre) of small (<20cm diameter) stone scattered more or less evenly over the surface. The overall area of the study (and therefore the distance to the target) was constrained by the physical environment.

### Data collection

The target was supplied with a list of random integer numbers generated using Microsoft Excel with a minimum value of 1 and a maximum value of 5; each number corresponded to a hiding location. Each number was different from the last to ensure that the target had to relocate after each approach by an observer. To mimic typical poacher behaviour, the target was instructed to assume a crouched position during the trial and to avoid any movement or making any noise.

**Fig 1:**
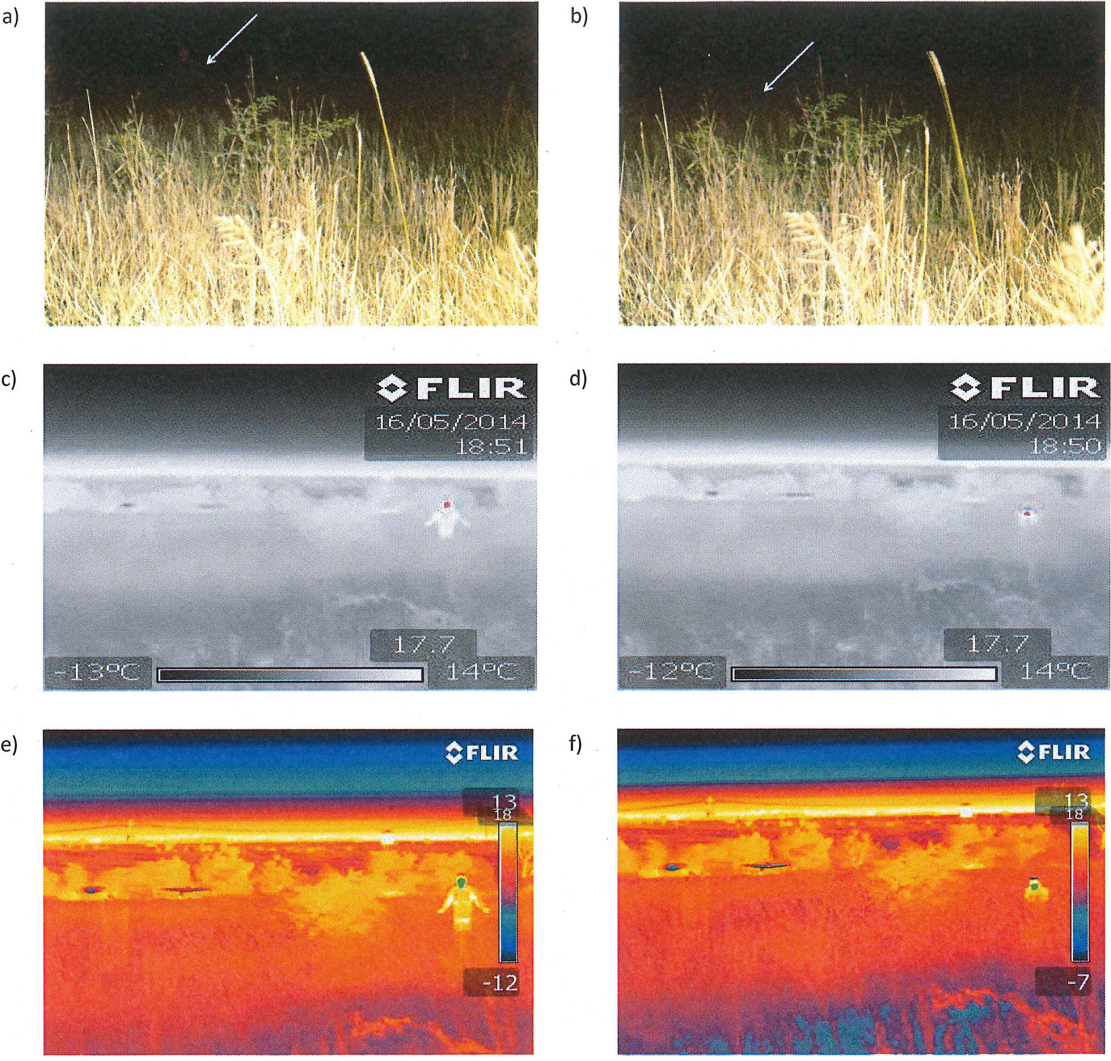
View of poacher from 20 m in field study area at hiding location 1: (a-b) illumination by an LED flashlight (technology 1) captured with standard digital SLR camera (Canon 7D) mounted on a tripod with a 30 s exposure to replicate, as nearly as possible, what was visible to observers with the naked eye (poacher location is indicated with a white arrow); (c-d) images from low-specification infrared thermography device (Flir i7; technology 2); (e-f) images from high-specification infrared thermography device (Flir T620; technology 3). In all cases, the images on the left are with the poacher standing up so location can be seen more clearly and the images on the right are with the poacher hiding in the crouched position that was adopted for all trials. IRT settings were as per methods and all IRT images shown are as they appeared on the IRT device in the field (a bush camp and powerlines are also visible in all images).

Over successive nights, a total of 27 observers participated in the study. Observers were second year biology undergraduates participating in a field course at the study site. Observers were all volunteers, who were fully briefed beforehand and who all gave informed verbal consent to authors AH, AG and RR before undertaking the study. All observers were over 18, not vulnerable adults and the procedure was solely observational with no interventions. Consequently, following the gatekeeper procedure of the University of Gloucestershire’s Ethics Panel, the study did not require formal ethical clearance. However, ethical clearance was obtained through the Chair of the University’s Research Ethic Committee. Observers took part on their own (i.e. not in teams) and were asked not to pass on any information about the study or experimental set-up to others. Each observer was walked to the study site without artificial light to allow their vision to adapt to the darkness (the walk and any waiting in the field prior to data collection starting took less than 20 minutes in all cases). Once at the start of the study site, each observer walked slowly down the approach path. Observers made three such walks in total each using a different method of detecting the poacher: (1) light from a very powerful flashlight (Model X21, LED Lenser, 1068 lumen power and 500m beam), (2) FLIR i7 low-specification IRT (hereafter LIRT); and (3) FLIR T620 high-specification IRT (hereafter HIRT) (Fig 1). The order of each of the three trials for each observer was randomised and the position of the target was also changed randomly (as described above) following each trial. As they walked down the approach path searching for the target, observers were required to indicate to one of the research team (SD, co-author, who was following the observer at a distance of a few metres and illuminating their path with a low power flashlight for safety) when they first thought they saw the target and to indicate the location by pointing. Then, following an identical search procedure, each observer continued walking (without assuming they were correct in their first location) until they were positive that they saw the target. In both cases, the distance along the approach path was noted by the accompanying member of the research team. The observer then indicated where they thought the poacher was and the poacher stood up so that the accuracy of the observer’s first and positive detections could be verified. This was a double-blind protocol, with only the target having location knowledge. Neither the observer, nor any member of the research team, knew where the target was at any point during the study.

Before each observer took part in the research, they were all complete novices with using IRT equipment. They were provided with very basic training in the use of the two IR devices (covering how to hold the device and what the colour screen showed when a hotpoint was detected). If observers used glasses or contact lenses for long range vision then they were required to use them for the study. They were also given a short and standardised oral briefing on the procedure for approaching study site and searching for the target:

“There is a “poacher” hidden somewhere to the left of the track. Starting at this start point, which is marked with two flags and where we are currently standing, walk straight ahead while looking for the poacher to the left of the track using whatever device you have been allocated. Always walk on the track. You will be accompanied by a researcher throughout and when you first think you see the poacher, you should stop and inform that researcher where you think the poacher is. Then continue walking along the track, looking in the same direction as previously. When you are absolutely positive that you can see the poacher, you should stop and inform the researcher, indicating exactly where you think the poacher is.”

Trials started 1–2 hours after sunset across three two-hour periods on three consecutive nights to control as far as possible for changes in moonlight (full moon was on the second night). After each observer’s three trials were completed, the distance between the point at which the observer detected the poacher as a first detection and the point at which the observer detected the poacher as a positive detection were calculated. This used the linear distance along the approach path (recorded as above) and the known distance between the hiding point used for that trial once this was revealed by the target and was calculated using trigonometry. These detection distances were calculated for each of the three trials for each observer. Each observer was then also separately tested for visual acuity (using a standard Landolt C Visual Acuity chart positioned at 4m in shaded daylight) and for colour blindness (using a set of Isihara colour palettes).

### Data analysis

To establish whether there was an association in the number of correct versus incorrect identifications of the target’s location according to technology type, two separate 3X3 chi square goodness of fit tests were conducted. In both cases, there were three test conditions (flashlight, LIRT, HIRT) and three possible outcomes (target location correctly identified, target location incorrectly identified, observer failed to see or identify the target at any time in the trial). The first test used the detection accuracy data from the point at which the observers first thought they saw the target, while the second test used data from the point at which the observer said they were positive they saw target. Then, to analyse whether there was a statistically significant difference in detection distances for all cases when target location had been identified correctly, a two-way repeated measures ANOVA was undertaken on the repeated measurements of poacher location. The two fixed factors were: (1) detection type (two levels–first detection and positive detection); and (2) technology type (three levels–flashlight, LIRT, HIRT). The interaction term was also calculated, and the Greenhouse-Geisser correction was used to compensate for the lack of sphericity in the dataset. Bonferroni post-hoc testing was undertaken to establish the pairwise differences in detection distances for the three different technology types (flashlight, LIRT, HIRT). This allowed for family-wise errors due to multiple tests being performed on the same dataset. The Bonferroni-adjusted critical significance level was α = 0.05. Using a repeated measures framework allowed for inter-individual differences in ability to locate the poacher, speed of walking, and so on, thereby increasing the robustness of the analysis.

Despite careful study design and analysis, it remained possible that the ease with which the poacher could be seen was not consistent between the five target locations (for example, if the locations themselves differed slightly in vegetation around the hiding site and along the line of sight between the hiding site and locations on the approach path). This could confound the results if, by chance, there was a bias in which of the five hiding locations corresponded most often with trials using one specific technology (i.e. if it was relatively easier to see the target at hiding location 1, and this location was, by chance, over-represented for HIRT, that could artificially inflate the apparent effectiveness of HIRT). Accordingly, to test whether there was a difference in detection distance between the hiding locations for each of the three technology types, three one-way ANOVAs were conducted with detection distance as the dependent variable and hiding locations as the independent variable. All data were analysed using SPSS v21 and α = 0.05 was used as the critical significance value in all analyses. All relevant data are within the paper and its Supporting Information files.

## Results

### Hiding location differences

Based on a critical significance of α = 0.05, no significant differences in detection difference between the five different hiding locations for any of the three technologies were found (one-way ANOVA: flashlight F = 1.151, P = 0.346; LIRT F = 1.554, P = 0.204; HIRT F = 1.050; P = 0.406; d.f. = 4 in all cases; given the non-significant results for the full models, post-hoc testing was not undertaken). This indicates that different hiding locations were very similar with regards to the ease of detecting the poacher, and that any random underlying associations between hiding location and technology type would not have confounded results.

### Accuracy of poacher detection

**Fig 2:**
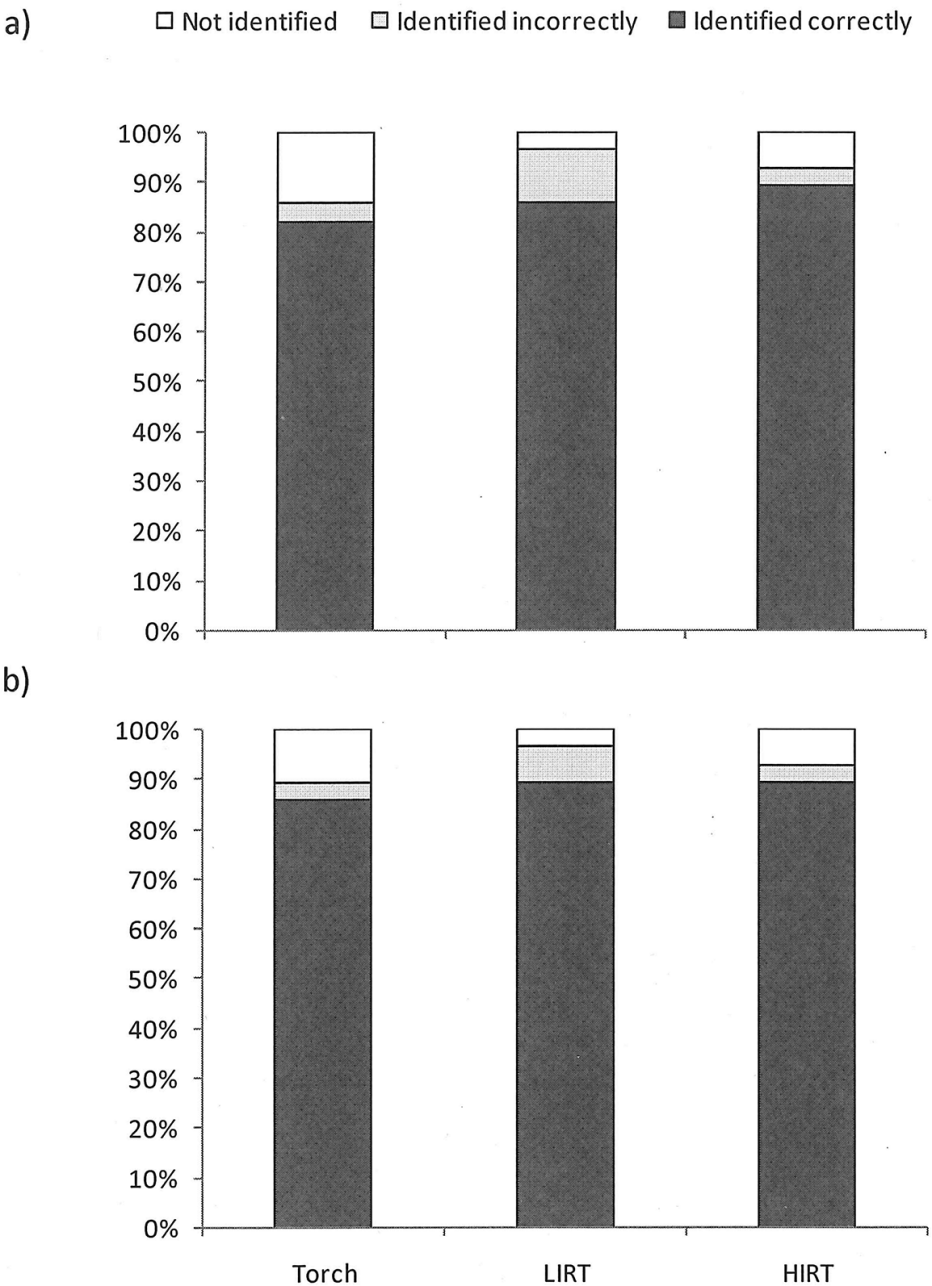
Accuracy of identifying poacher location using different technologies: (a) accuracy at first detection; (b) accuracy at positive detection.

Generally, target location was identified accurately using all three technologies. Overall, accuracy of correct detections (i.e. the percentage of correct first and correct positive detections across all trials for all observers) was 86.9%, with each technology having high accuracy of detection (83.9% accuracy with a flashlight, 87.5% with LIRT, and 89.3% with HIRT). Occasions where the target’s location was not identified correctly were split approximately equally between trials where the location was not identified at all (7.7%) and trials where it was identified incorrectly (5.4%) (Fig 2). Across all observers and all trials accuracy was slightly better for positive detections (88%) than for first detections (85%). There was no significant association between the accuracy of detecting the target and technology type for either first detection (Fig 2A; chi square test for association: χ^2^ = 3.680, d.f. = 4, P = 0.451) or positive detection (Fig 2B; χ^2^ = 1.560, d.f. = 4, P = 0.822). These results suggest that the type of technology used does not affect the *accuracy* of detection of poachers.

### Detection differences

**Fig 3:**
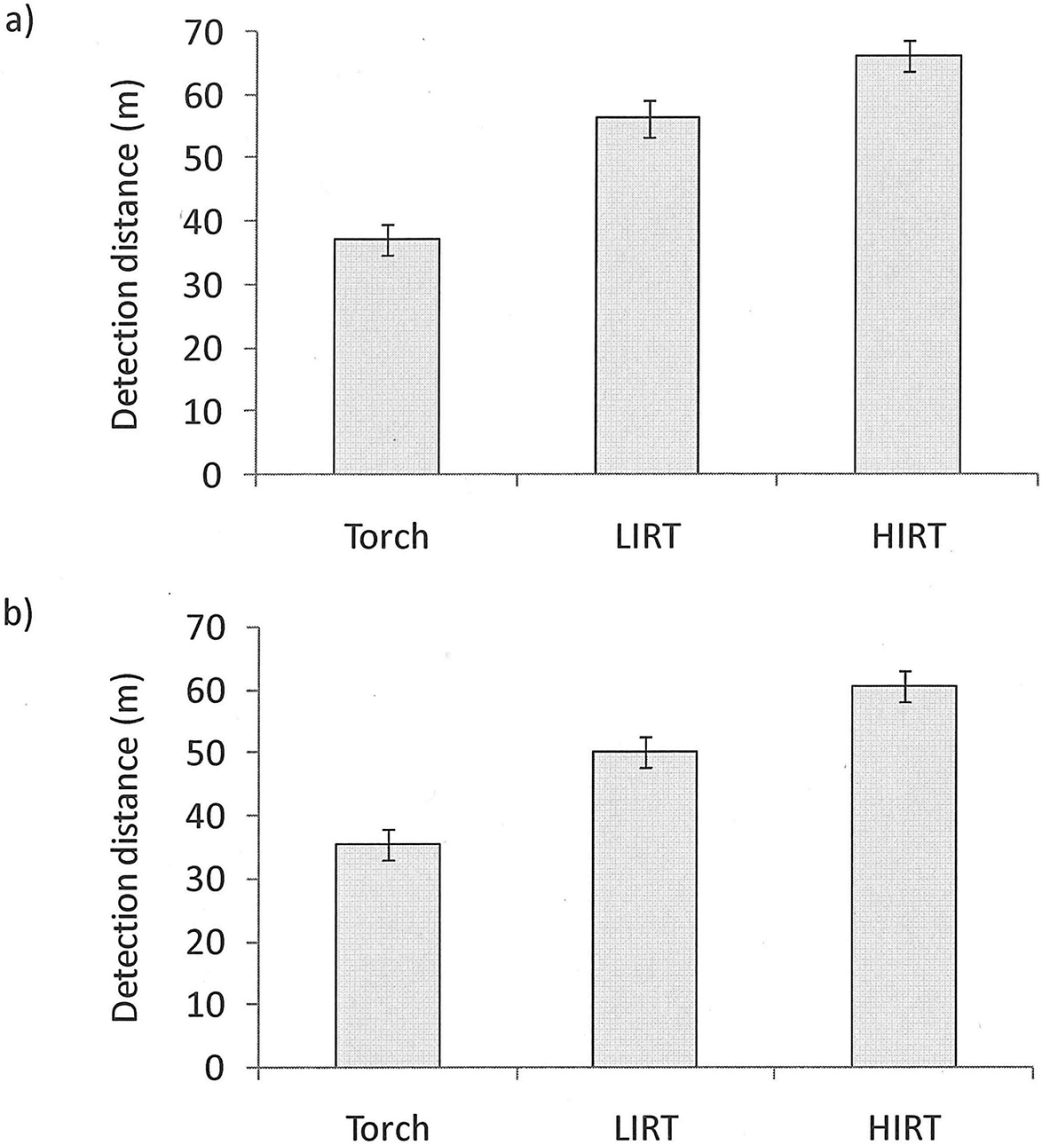
Mean distance (m) for accurate detection of poacher location using different technologies: (a) distance between observer and poacher at first detection; (b) distance between observer and poacher at positive detection. Error bars represent standard error.

For both first and positive detections, mean distance for correct detection were lowest (i.e. observers had to get closest to the target) for the flashlight (first detection = 37.3m ± 2.4m SEM; positive detection mean = 35.4m ± 2.4m SEM). Correct detection was possible at further distance when the LIRT was used (first detection mean = 57.1m ± 2.9m SEM; positive detection mean = 50.3m ± 2.4m SEM) and further still when the HIRT was used (first detection mean = 68.3 ± 3.2 SEM; positive detection mean = 62.9 ± 2.2 SEM) (Fig 3). Detection distances were influenced significantly both by technology type (flashlight, LIRT, HIRT) and detection type (first versus positive) when analysed using a repeated measure ANOVA to allow for inter-individual differences. The interaction between technology type and detection type was also significant (Table 1). In terms of technology type, Bonferroni post-hoc testing showed that both first and positive detection distances were significantly better for both IRT devices relative to flashlight light, but the IRT devices themselves did not differ (Table 1). In terms of the detection type the distance between the observer and the target was significantly greater at the point of first detection than at the point of positive detection (Table 1). Interestingly, though, the magnitude of the difference in detection distances between first and positive identification varied between technology types, which is why the interaction term was significant. This significant interaction arose because there was very little difference in the detection distances for first and positive target detection when using a flashlight (mean difference = 1.9m + 0.9m SEM), which suggests that observers using a flashlight did not get much of an early warning of poacher location. For both IRT technologies, however, the point of positive detection was, on average, 6.2m ± 1.4m SEM for LIRT and 5.7m ± 1.5m SEM for HRT nearer to the target than the point of first detection, such that using an IRT gave an earlier warning of possible poacher presence than did a flashlight.

**Table 1:** Repeated measures ANOVA on detection differences for a poacher in South African bushveldt using three different technologies (SS_M_, SS_E_ and SS_T_ = Sum of Squares for Model, Error and Total respectively).

ANOVA Term	F	d.f.	P	SS_M_	SS_E_	Partial R^2^	Details
Detection type (first versus positive detection)	17.122	1	0.001	635	659	0.031	Distance between observer and poacher smaller for positive detection than tentative
Technology type (torch versus LIRT versus HIRT)	25.725	1.952	<0.001	14006	4906	0.681	Post-hoc: torch vs LIRT and torch vs HIRT <0.001; LIRT vs HIRT = 0.228
Interaction	5.436	1.916	0.010	102	272	0.005	Smaller difference between positive and first detection differences for torch than LIRT/HIRT

Partial R^2^ calculated as SS_M_/ SS_T_. The Greenhouse-Geisser correction was used to compensate for the lack of sphericity. Consequently, degrees of freedom are calculated by multiplying the actual degrees of freedom by the estimate of sphercity. Overall model: SS_T_ = 20580; R^2^ = 0.716.

## Discussion

We found support for the hypothesis that thermal imaging has a clear advantage over a flashlight in terms of the distance at which the target could be detected. Overall, HIRT added 11.2m to mean first detection distance of LIRT, which gave a 19.8m advantage over a flashlight. This meant that detection distances almost doubled using HIRT relative to traditional flashlight technology. Though both IRT devices were capable of detecting the poacher at significantly greater distances than a flashlight, we found no evidence to support the hypothesis that more expensive equipment would provide a greater detection distance. We found that there was no statistically significant difference in detection distance between LIRT and HIRT. Furthermore, the accuracy of all devices was not significantly different, in other words although a flashlight user may need to be far closer to a target than an IRT user, both users can be equally confident that a positive detection really is a poacher. IRT technology will not likely replace flashlights, but the increase in range of detection could prove to be invaluable for many game reserves.

Previous studies have investigated the use of thermal imaging cameras as a tool for anti-poaching by either mounting them on to unmanned aerial vehicles (UAVs) [20] or deploying them on weather balloons [34]. Although both methods are feasible, they have major limitations. Firstly, UAVs are constrained by short flight times (typically 30–40 minutes) and local aviation regulations. Secondly, the presence of obstacles such as power lines might cause problems, especially at night where visibility is limited. Thirdly, the effectiveness of cameras mounted on UAVs is limited by altitude. An altitude of 100-180m has been suggested [20] for suitable detection of poachers, but flying this low incurs greater risks of the plane being detected from the ground and potentially disrupting animals. The proposed system by Tan et al. (2013) [34] involves deploying weather balloons fitted with IRT and that are able to alert rangers when a threat is detected. Weather balloons can be flown for up to 12 hrs, however, once again the on-board IRT technology is most optimal at an altitude of 150m or less, which would allow poachers to detect them easily from the ground. Ol Pejeta, a Kenyan wildlife reserve, has already put IRT technology to use through a UAV-mounted device. The device costs 70,000 USD and is fitted with a live streaming high definition camera that has a high powered zoom for daytime and infrared thermal imaging for night-time operations. The UAV covers 50 miles in 1.5 hr and is flown 3–4 times per day. This acts as a deterrent to poachers [34], but is extremely costly and behind the financial reach for small reserves. Moreover, with any remote option, patrols may require considerable time to reach the suspected poaching location after being alerted. Given that the HIRT did not give a significant increase in detection distance over the LIRT yet costs over ten times the price, the HLRT would not seem to be justified solely because of detection performance for anti-poaching patrols. This study suggests that game reserves would be better (or at least, no worse) to opt for the LIRT on the basis of cost alone if they were to invest in IRT technology for use by foot patrols for detecting poachers. Additionally, feedback from users in this study indicated that the LIRT was easier to use in field conditions because of its lighter weight, ergonomic design and simplified screen output. However, a drawback of both handheld IRT devices was also highlighted by the study: several users noticed that the IRT screen seriously disrupted night vision and could cause the user to become disoriented and cause stumbling as the user could no longer clearly see the approach path. It might, however, be possible to reduce this problem using a red filter over the display. It was also noted by the target that the IRT screen has the effect of making the illuminated user’s face visible to poachers, although a flashlight beam may be visible to poachers at a greater distance.

This study suggests that IRT could benefit wildlife reserves for detecting poachers *in situ*. However, reserves would be prudent to analyse the increase in poacher prevention/control in their specific environment before investing in this technology. For example, this study shows that IRT is highly effective in grassland, but in areas with extensive thick scrub it might not be so effective. It may also be possible for poachers to evade detection by adopting certain hiding positions or wearing certain clothing, and learning to avoid thermal detection would compromise IRT as a search tool. The potential problem false positives should also be considered in different environments, especially those with objects likely to have a strong thermal image that could be confused for a poacher, for example, a rocky environment. Furthermore, we have only tested an albeit realistic scenario where searchers are aware of the general target location, but the usefulness or otherwise of using IRT to scan larger areas as part of a vehicle patrol needs to be tested under realistic field conditions. However, even in more complex environments it is likely that IRT would provide enough of an advantage to make it a worthwhile investment. Reserves considering IRT might be best to invest in a number of less expensive IRT units, with multiple units distributed among series of patrols.

## Supporting Information

S1 File Ethical Clearance. (PDF)

S1 Table Accuracy and detection distances. The distances along the transect at which subjects detected the hidden target “poacher” (both first, preliminary, detection and positive detection) for all subjects using flashlight, LIRT and HIRT. The target’s location is used to trigonometrically calculate the actual detection distance from the detection distance along the transect. The accuracy of detection is also given (0 = wrong; 1 = correct).
(XLSX)
