# Supplementary material for: Can Handheld Thermal Imaging Technology Improve Detection of Poachers in African Bushveldt?
Source: PLoS One. 2015 Jun 25;10(6):e0131584. doi: 10.1371/journal.pone.0131584 (PMC4481516; doi:10.1371/journal.pone.0131584)
Supplement: S1 File — (PDF) [file pone.0131584.s001.pdf]

UNIVERSITY OF GLOUCESTERSHIRE
at Cheltenham and Gloucester

Academic Registry
The Park
Cheltenham
GL50 2RH
Tel: 01242 714700

Prof Adam Hart
School of Natural and Social Sciences
Francis Close Hall
University of Gloucestershire

5 January 2015

Dear Adam

Thank you for the enquiry regarding ethics approval for your project exploring whether handheld thermal imaging technology can improve the detection of poachers in African bushveldt.

We would normally deal with projects of this kind, staff managed and low risk with minimal ethical concerns, through the gatekeeper system outlined in the university's publication *Research Ethics: A Handbook of Principles and Procedures*. In this case, the project would be reviewed by the Faculty's Associate Dean Research/Head of Research unit or equivalent. We recognise, however, where a low risk activity is developed in the field to take account of an opportunity that arises then some flexibility is necessary.

We note that a full risk assessment had been carried out for the trip this project was part of and that the participants in the project were not asked to do anything that they would not otherwise have been expected to do in the course of the trip, except that they were using hand held thermal imaging equipment.

On this basis, and noting the provisions outlined in the methods section of the draft paper 'Can handheld thermal imaging technology improve detection of poachers in African bushveldt?', I can confirm that the project is fully consistent with the University of Gloucestershire's research ethics provisions.

This note stands in lieu of written approval through the usual gatekeeper process.

Best wishes

Dr Malcolm MacLean
Chair- Research Ethics Committee

cc. Dr Sharon Brookshaw, REC Officer
